# Circulating glucagon-like peptide-1 increases in response to short-term overfeeding in men

**DOI:** 10.1186/1743-7075-10-33

**Published:** 2013-04-08

**Authors:** Danny Wadden, Farrell Cahill, Peyvand Amini, Edward Randell, Sudesh Vasdev, Yanqing Yi, Jon Church, Guang Sun

**Affiliations:** 1 Division of Medicine, Faculty of Medicine, Health Sciences Center, Memorial University of Newfoundland, 300 Prince Phillip Drive, St. John’s, NL, A1B 3V6, Canada; 2 Discipline of Laboratory Medicine, Health Sciences Center, Memorial University of Newfoundland, 300 Prince Phillip Drive, St. John’s, NL, A1B 3V6, Canada; 3 Memorial University of Newfoundland, 300 Prince Philip Drive, St John’s, Newfoundland, A1B 3V6, Canada

**Keywords:** Overfeeding, GLP-1, Nutritional regulation, Obesity, Diabetes

## Abstract

**Background:**

Glucagon-like Peptide-1 (GLP-1) is an incretin hormone secreted from the gastrointestinal tract that facilitates the glucose-dependent insulin response. Additionally, GLP-1 is thought to be involved in energy homeostasis. Currently little is known about GLP-1’s responsiveness to an energy surplus, a fundamental cause of obesity and diabetes. Our objective was to examine the response of serum GLP-1 to short-term (7 day) overfeeding in young men.

**Methods:**

Seventy-two young men from the Canadian province of Newfoundland were recruited for the study. For 7-days, the subjects consumed 70% more calories than required at baseline.

Various measurements including: anthropometrics, body composition, markers of glucose/lipid metabolism and serum total GLP-1, were taken at a fasted state before (day 1) and after (day 8) the challenge. Paired t-test analyses were used to assess the change in variables after the overfeeding period. Additionally, the relationship between serum GLP-1 and the measured variables at baseline and change due to overfeeding were analyzed.

**Results:**

Serum GLP-1 was significantly increased in all groups in response to the 7-day energy surplus, indicating the increase was independent of adiposity status. There was no significant difference in fasting GLP-1 at baseline between the normal weight and overweight/obese groups. At baseline, GLP-1 concentration negatively correlated with HDL-cholesterol and positively correlated with triacylglycerols and markers of insulin resistance in the overweight/obese group. Also GLP-1 was negatively correlated with change in percent gynoid fat in the overweight/obese subjects. Percent change in GLP-1 was negatively associated with percent change in gynoid fat in the normal weight group and positively associated with percent change in cholesterol in the overweight/obese group. Percentage change of circulating triacylglycerols was positively associated with percent change in GLP-1 in both adiposity groups.

**Conclusion:**

Our findings showed that GLP-1 serum concentration is not a significant factor in determining obesity status. The increase of GLP-1 in all subjects regardless of obesity status, suggest GLP-1 serves as a protective role, counteracting energy surplus.

## Introduction

Hormones secreted from the gastrointestinal (GI) tract play an important role as peripheral signals of energy homeostasis and are thought to be involved in the development of obesity and diabetes [1]. Glucagon-like peptide-1 (GLP-1) is a 30-amino acid peptide hormone secreted from the L-cells of the distal ileum, but is also present in the central nervous system [2]. GLP-1 is a product of the GCG gene and is formed due to tissue-specific post-translational modification of proglucagon. The hormone is released into circulation in response to food intake; the larger the meal size, the greater the GLP-1 response [3,4]. Therefore during the fasted state, GLP-1 is low (but still detectable) however the concentration rises postprandially. Active GLP-1 has a very short half-life of ~2 minutes as it is degraded by dipeptidyl peptidase-IV (DPP-IV) [5,6].

GLP-1 binds to its receptors present in a variety of tissues and elicits a number of responses. For one, GLP-1 is designated an incretin hormone, facilitating the glucose-dependent release of insulin from the pancreatic beta-cells [7,8]. Also it has been shown to decrease the secretion of glucagon in patients with type-I diabetes who have no beta-cell function but still exhibit the lowered plasma glucose via GLP-1 [9-11]. Other effects include, decreased appetite [12-14] and decreased gastric motility and secretion [15,16].

Due to its many metabolic-related effects, GLP-1 has been implicated in many chronic metabolic diseases. For example, both its incretin effect and suppression of glucagon secretion action are disrupted in type-II diabetes [17]. It has also been shown that intravenous (IV) infusion of GLP-1 acts to lower blood glucose concentration in type-II diabetic patients [18,19]. GLP-1 is also suggested to be involved in obesity as morbidly obese subjects show a decreased diurnal L-cell secretion [20]. Fasting GLP-1 has been found to be lower in diabetic and obese diabetic patients as compared to healthy controls [21,22] however, not all studies have consistent findings [23]. Additionally, the postprandial secretion of GLP-1 is inhibited in morbidly obese subjects, which is improved after weight loss [24,25]. It has been found that GLP-1 infusions reduce food intake in normal weight and obese subjects, regardless of diabetic status [13,14,26-28].

Recognizing that obesity is a result of a chronic positive energy imbalance, it has been shown that modification of food intake, including overfeeding, influence various gut hormone concentrations [29-32]. However, most human studies on GLP-1 have been performed using a cross sectional study design which may not reflect biological and clinical relevance. The dynamic process of an energy surplus will provide insights of the role of GLP-1 in the development of obesity and diabetes [33-35]. The objectives of the present study were therefore to investigate: a) the GLP-1 response to short-term overfeeding in young men; b) the difference in serum GLP-1 concentration in pre- and post- overfeeding between various adiposity groups; and c) the relationship of fasting GLP-1 with various obesity-related markers.

## Methods

### Subjects

Seventy-two healthy men (age 19–29) from Newfoundland and Labrador, Canada, participated in the present study. All subjects were of at least 3^rd^ generation Newfoundland descent and reported a stable body weight over the last six months. Participants had no serious endocrine, metabolic or cardiovascular diseases, nor were taking any medications affecting lipid/cholesterol metabolism. Informed and written consent were provided by each subject. Ethical approval was received from the Human Investigations Committee for the Faculty of Medicine, Memorial University, St. John’s, NL, Canada.

### Overfeeding

Overfeeding was completed following a protocol previously described by us [31,32,36]. To assess the metabolic and endocrine effects of a short-term energy surplus, participants were overfed, for one week (7 days), 70% more calories than what they would normally consume. A 7-day overfeeding period was chosen to ensure changes in metabolic parameters. To estimate daily energy requirements, three 24-hour recall interviews (2 on weekdays, 1 on weekend) and a 30-day dietary inventory were administered to the subjects. A food recall kit containing standard portion sizes was used in a face-to-face interview, assessing the food intake of the previous 24-hours. An average of the questionnaires was utilized to determine daily caloric requirements. The positive energy challenge was consistent with the typical North American diet: 50% carbohydrates, 35% fat, and 15% protein. Throughout 7-days at time 0900, 1200, and 1700, subjects consumed meals with caloric and nutritional content calculated using Food Processor SQL (version 9.5.0.0; ESHA Research, Salem, OR). A laboratory member was present through the duration of all meals. Average caloric content was 2969 kcal pre-overfeeding and 5471 kcal during overfeeding [29]. While participating in the study, subjects were requested to refrain from consuming additional calorie-containing beverages, drinking alcohol, or taking drugs/medications.

### Measured variables

Various physical, anthropometric and biochemical markers were assessed after a 12-hr fasting period, before and after the 7-day overfeeding period. These are outlined below:

#### Anthropometric measurements

During all anthropometric measurements subjects wore a light standardized hospital gown. Total body weight was assessed on a platform scale balance (Health O Meter, IL) and height was measured using a fixed stadiometer, to the nearest 0.1 kilogram and centimeter, respectively. Body mass index (BMI) was calculated by dividing the participant’s weight in kilograms by height in meters squared (kg/m^2^). Both waist and hip circumference were evaluated using a measuring tape to the nearest 0.1 centimeter.

#### Body composition measurements

Body composition was assessed using dual-energy X-ray absorptiometry (DXA Lunar Prodigy; GE Medical Systems, Madison, WI). The scan can differentiate between fat, lean, and bone mass, and can therefore determine percent body fat (%BF), trunk fat (%TF), android fat (%AF), and gynoid fat (%GF) [37] (method previously described by us [38]). Study subjects were classified as underweight, normal weight, overweight, and obese based on percentage body fat recommendations by Bray (for males aged 20–39: underweight= <8.0%, normal weight= 8-20.9%, overweight= 21–25.9%, or obese= >26.0%) [39].

#### Serum measurements

Venous blood was collected from subjects and after processing, serum was stored at −80°C. Serum total cholesterol, high-density lipoprotein cholesterol (HDL-c), triacylglycerols (TGs), and glucose concentrations were determined by Synchron reagents using an Lx20 clinical chemistry analyzer (Beckman Coulter Inc., CA, USA). The concentration of serum insulin was measured utilizing the Immulite 2500 immunoassay analyzer (Siemens, Los Angeles, CA). The homeostasis model assessment (HOMA) was used to estimate indices of pancreatic β-cell function (HOMA-β: [20 × insulin (mU/L)/(glucose (mmol/L) - 3.5)]) and insulin resistance (HOMA-IR: [insulin (mU/L) × glucose (mmol/L)/22.5)]). The Friedwald equation (total cholesterol – HDL-c – TG/2.2) was used to calculate low-density lipoprotein cholesterol (LDL-c). Serum total GLP-1 was measured in duplicate using enzyme-linked immunosorbent assay (ELISA) kits (EMD Millipore, St. Charles, MO). The intra-assay variation ranged from 3.4% to 4.2%, and the inter-assay variation (n = 4) was 5.7%.

### Statistical analysis

All data are presented as mean ± S.E. unless stated otherwise. Data not normally distributed were log-transformed (concentrations of fasting: serum GLP-1, triacylglycerols, insulin, HOMA-IR and HOMA-β) where appropriate. As well, all data was analyzed using SPSS (Version 19) and statistical tests were two-sided with significance set at a P-value of 0.05. Because of the small sample size (n=3) underweight subjects were combined with normal weight subjects (total n=30). Additionally, as there were a small number of overweight individuals (n=14), these subjects were combined with obese subjects (total n=42).

The differences between various markers of adiposity, insulin resistance, lipid metabolism, and fasting GLP-1 concentration before and after the overfeeding protocol, were assessed utilizing paired t-test analysis. Additionally a two-factor ANOVA (repeated measures) was used to examine the overfeeding-adiposity interaction of measured variables. One-way ANOVA (with Bonferroni post hoc tests) was utilized to assess the differences of variables between adiposity groups at baseline.

Spearman correlation analysis was completed to assess the relationship between a) baseline GLP-1 serum concentration and aforementioned variables at baseline, b) baseline GLP-1 serum concentration and change in the variables; c) change in serum GLP-1 and change in the variables; and d) baseline variables and change in serum GLP-1. Additionally partial correlative analysis was completed controlling for potential confounding factors.

## Results

### Pre- and post- overfeeding descriptive statistics

Biochemical and physical measurements prior-to and after the overfeeding challenge are presented in Table 1. Changes in anthropometrics, body composition and measures of glucose and lipid metabolism were previously described by us [31,32,40]; nevertheless, it was evident that the one-week overfeeding challenge increased body weight, adiposity, serum lipids, insulin and insulin resistance. After a one week energy surplus, circulating GLP-1 concentration rose in the cohort. The pre- and post- overfeeding fasting concentrations of GLP-1 were increased from 36.84±3.16 pmol/L at baseline to 42.39±3.18 pmol/L, after overfeeding. The average percent change in GLP-1 through the overfeeding study was 24.10±5.85%. However based on 2-way ANOVA repeated measure analysis, there was no significant difference between normal weight and overweight/obese subjects for the increase in serum GLP-1 (P=0.590). Furthermore, there was no significant difference between the normal weight and overweight/obese group for fasting GLP-1 concentration at baseline (t-test: P=0.876).

**Table 1:** **Physical and biochemical characteristics of subjects at baseline and in response to 7-days of overfeeding **^**a,b**^

	**Entire cohort (n = 72)**				
	**Day 1**	**Day 8**	***p-value***	**Change (Δ) from baseline**	**Average percent change from baseline (%)**
Age	23.11 ± 0.37	N/A	-	N/A	N/A
Height (cm)	179.18 ± 0.72	N/A	-	N/A	N/A
Weight (kg)^b^	80.92 ± 1.81	83.13 ± 1.87	<0.001	2.21 ± 0.16	2.74 ± 0.20
BMI (kg/m^2^)^b^	25.27 ± 0.56	25.96 ± 0.58	<0.001	0.69 ± 0.05	2.74 ± 0.20
Percent body fat	22.41 ± 1.05	22.69 ± 1.00	0.051	0.28 ± 0.14	2.65 ± 0.87
Percent trunk fat	25.15 ± 1.17	25.52 ± 1.10	0.088	0.38 ± 0.21	3.25 ± 1.10
Percent android fat^b^	28.64 ± 1.38	29.44 ± 1.35	<0.001	0.80 ± 0.21	4.77 ± 1.37
Percent gynoid fat	27.27 ± 1.03	27.50 ± 0.99	0.221	0.23 ± 0.19	1.80 ± 0.89
Total cholesterol (mmol/L)^b^	4.50 ± 0.10	4.74 ± 0.10	0.007	0.23 ± 0.08	6.33 ± 1.88
HDL cholesterol (mmol/L)^b^	1.31 ± 0.03	1.41 ± 0.03	<0.001	0.10 ± 0.02	9.08 ± 1.88
Total cholesterol:HDLc ratio^b^	3.58 ± 0.10	3.47 ± 0.10	0.020	−0.10 ± 0.04	−2.11 ± 1.12
LDL cholesterol (mmol/L)	2.72 ± 0.08	2.75 ± 0.08	0.639	0.03 ± 0.06	2.88 ± 2.17
Triacylglycerols (mmol/L)^b^	1.08 ± 0.06	1.44 ± 0.16	0.005	0.35 ± 0.15	44.74 ± 21.54
Glucose (mmol/L)	5.10 ± 0.06	5.11 ± 0.06	0.905	0.01 ± 0.07	0.75 ± 1.31
Insulin (pmol/L)^b^	68.54 ± 8.27	86.84 ± 7.78	<0.001	18.29 ± 6.98	55.02 ± 9.53
HOMA-IR^b^	2.38 ± 0.34	2.92 ± 0.29	<0.001	0.54 ± 0.29	59.71 ± 10.77
HOMA-β^b^	114.57 ± 8.85	158.38 ± 12.38	<0.001	43.80 ± 9.30	50.60 ± 8.01
Serum GLP-1 (pmol/L)^b^	36.84 ± 3.16	42.39 ± 3.18	<0.001	5.55 ± 1.57	24.10 ± 5.85

^a^ Values are mean ± SE. Homeostasis model assessment of insulin resistance (HOMA-IR) and of B cell function (HOMA-β); GLP-1, Glucagon-like Peptide-1; N/A, not applicable.

^b^ Significant difference present between pre- and post- overfeeding (paired t-test; SPSS 19.0).

### Baseline correlations of GLP-1 with body composition and markers of lipid/glucose metabolism

Table 2 characterizes the baseline relationships between GLP-1 and the markers of adiposity, and lipid/glucose metabolism, utilizing Spearman correlation analysis. In the entire cohort, fasting GLP-1 concentration was negatively associated with HDL-c concentration and positively associated with triacylglycerol concentration. Also a positive relationship was evident between GLP-1 and the ratio of total cholesterol to HDL-c. When the cohort was grouped based on adiposity status (based on Bray recommendations), no relationships were found between GLP-1 concentration and the measured variables in the normal weight group. However, in the overweight/obese group, GLP-1 concentration remained significantly correlated with HDL-c, triacylglycerol concentration, and the ratio of total cholesterol to HDL-c. Interestingly, in the overweight/obese, a positive relationship was found between GLP-1 concentration and insulin, HOMA-IR, HOMA-β, and percent gynoid fat.

**Table 2:** **Spearman correlations of baseline variables related to baseline fasting serum GLP-1 concentration **^**a,b**^

	**All Subjects (n = 72)**		**Normal weight (n = 30)**		**Overweight+Obese (n = 42)**	
	***r***	***P***	***r***	***P***	***r***	***P***
Weight (kg)	0.052	NS	−0.197	NS	0.175	NS
BMI (kg/m^2^)	−0.006	NS	−0.218	NS	0.117	NS
Percent body fat	0.086	NS	−0.027	NS	0.186	NS
Percent trunk fat	0.098	NS	−0.059	NS	0.238	NS
Percent android fat	0.079	NS	−0.075	NS	0.216	NS
Percent gynoid fat	0.141	NS	−0.021	NS	0.328	0.036
Total cholesterol	0.103	NS	0.265	NS	−0.037	NS
HDL cholesterol	−0.357	0.002	0.141	NS	−0.582	<0.001
Total cholesterol:HDLc ratio	0.383	0.001	0.182	NS	0.489	0.001
LDL cholesterol	0.077	NS	0.286	NS	−0.065	NS
Triacylglycerols	0.412	<0.001	0.232	NS	0.526	<0.001
Glucose	0.157	NS	0.039	NS	0.191	NS
Insulin	0.216	NS	0.001	NS	0.394	0.010
HOMA-IR	0.207	NS	0.005	NS	0.375	0.014
HOMA-β	0.205	NS	−0.074	NS	0.353	0.022

^a^ P<0.05 (IBM SPSS Statistics 19).

^b^ Homeostasis model assessment of insulin resistance (HOMA-IR) and of β cell function (HOMA-β); NS, non-significant. Subjects were classifed based on adiposity recommendations by Bray as normal weight (8–20.9%), overweight (21–25.9%), or obese (>26%).

Due to the possible confounding effect of gynoid fat on HDL-cholesterol, triacylglycerols, and markers of insulin metabolism, partial correlations were performed controlling for gynoid fat and all analyses were repeated (data not shown). All of the aforementioned relationships remained significant except for the relationship between GLP-1 and HOMA-β in the overweight/obese group (r = 0.300, P = 0.053).

### Correlations between baseline GLP-1 with percent change in body composition and markers of lipid/glucose metabolism

The correlative data between fasting baseline GLP-1 concentration and percent changes in the markers of adiposity, and lipid and glucose metabolism were also observed. In the entire cohort, no relationships were present between circulating GLP-1 and changes in any of the measurements. However when subjects were grouped according to adiposity, a negative correlation was revealed between GLP-1 concentration and change in percent gynoid fat (r = −0.349, P=0.025) in the overweight/obese group.

### Correlations between percent change in GLP-1 with percent change in body composition and markers of lipid/glucose metabolism

Presented in Table 3 is the correlative data between percent change in GLP-1 concentration and percent change in markers of adiposity, and markers of lipid/glucose metabolism. In the entire cohort, change in GLP-1 was significantly positively associated with percent change in HDL-c, and triacylglycerols. When split based on adiposity status, percent change in GLP-1 was positively correlated with percent change in triacylglycerols and negatively correlated with percent change in gynoid fat in the normal weight group. However, in the overweight/obese percent change in serum GLP-1 was significantly positively associated with percent change in total cholesterol and triacylglycerols.

**Table 3:** **Spearman correlations of changes in variables related to change in fasting serum GLP-1 concentration **^**a,b**^

	**All subjects (n = 72)**		**Normal weight (n = 30)**		**Overweight+Obese (n = 42)**	
	***r***	***P***	***r***	***P***	***r***	***P***
Weight (kg)	0.222	NS	0.354	NS	0.137	NS
BMI (kg/m^2^)	0.223	NS	0.348	NS	0.142	NS
Percent body fat	0.122	NS	0.168	NS	0.087	NS
Percent trunk fat	0.126	NS	0.221	NS	0.021	NS
Percent android fat	0.206	NS	0.286	NS	0.066	NS
Percent gynoid fat	−0.061	NS	−0.384	0.036	0.243	NS
Total cholesterol	0.219	NS	0.117	NS	0.334	0.030
HDL cholesterol	0.258	0.029	0.321	NS	0.188	NS
Total cholesterol:HDLc ratio	−0.005	NS	−0.171	NS	0.191	NS
LDL cholesterol	0.007	NS	−0.085	NS	0.195	NS
Triacylglycerols	0.543	<0.001	0.573	0.001	0.533	<0.001
Glucose	0.131	NS	0.226	NS	0.099	NS
Insulin	0.218	NS	0.187	NS	0.271	NS
HOMA-IR	0.212	NS	0.198	NS	0.229	NS
HOMA-β	0.127	NS	0.024	NS	0.174	NS

^a^ P<0.05 (IBM SPSS Statistics 19).

^b^ Homeostasis model assessment of insulin resistance (HOMA-IR) and of β cell function (HOMA-β); NS, non-significant. Subjects were classifed based on adiposity recommendations by Bray as normal weight (8–20.9%), overweight (21–25.9%), or obese (>26%).

### Correlations between percent change in GLP-1 with baseline body composition and lipid/glucose metabolism variables

Additionally we wanted to assess the relationship between baseline variables and percent change in GLP-1 (Table 4). In the entire cohort, baseline weight and BMI were positively correlated with percent change in GLP-1 while baseline GLP-1 was negatively correlated with percent change in GLP-1. The same relationships were found significant in the normal weight group, when the cohort was grouped based on adiposity. However, only the negative relationship with baseline GLP-1 remained significant in the overweight/obese group.

**Table 4:** **Spearman correlations of baseline variables related to change in fasting serum GLP-1 concentration **^**a,b**^

	**All subjects (n = 72)**		**Normal weight (n = 30)**		**Overweight+Obese (n = 42)**	
	***r***	***P***	***r***	***P***	***r***	***P***
Weight (kg)	0.251	0.033	0.391	0.033	0.162	NS
BMI (kg/m^2^)	0.295	0.012	0.446	0.013	0.188	NS
Percent body fat	0.130	NS	0.272	NS	0.109	NS
Percent trunk fat	0.139	NS	0.307	NS	0.104	NS
Percent android fat	0.127	NS	0.278	NS	0.077	NS
Percent gynoid fat	0.068	NS	0.239	NS	−0.074	NS
Total cholesterol	−0.112	NS	−0.255	NS	−0.030	NS
HDL cholesterol	−0.085	NS	−0.291	NS	0.095	NS
Total cholesterol:HDLc ratio	−0.012	NS	0.066	NS	−0.082	NS
LDL cholesterol	−0.118	NS	−0.179	NS	−0.115	NS
Triacylglycerols	−0.151	NS	−0.226	NS	−0.110	NS
Glucose	0.016	NS	−0.122	NS	0.124	NS
Insulin	0.135	NS	0.079	NS	0.074	NS
HOMA-IR	0.135	NS	0.059	NS	0.083	NS
HOMA-β	0.158	NS	0.248	NS	0.063	NS
GLP-1	−0.416	<0.001	−0.484	0.007	−0.396	0.009

^a^ P<0.05 (IBM SPSS Statistics 19).

^b^ Homeostasis model assessment of insulin resistance (HOMA-IR) and of β cell function (HOMA-β); NS, non-significant. Subjects were classifed based on adiposity recommendations by Bray as normal weight (8–20.9%), overweight (21–25.9%), or obese (>26%).

## Discussion

In the current investigation we examined the response of circulating GLP-1 to a 7-day energy surplus in 72 young men of Newfoundland descent. The most notable finding was that GLP-1 concentration significantly increased in response to the overfeeding challenge. The rise in GLP-1 concentration was independent of adiposity status as the increase of GLP-1 was present in normal weight and overweight/obese groups. GLP-1 has been shown to have beneficial effects as it facilitates the glucose-dependent insulin response, lowers glucagon secretion, and induces satiation [7-14]. Thus current literature suggests GLP-1 secretion would increase in response to a positive energy challenge, counteracting the response and potentially acting as a protective mechanism (cessation of appetite/regulation of insulin secretion). Though this may be the case, studies regarding circulating GLP-1 and overfeeding in humans are few in number, and vary largely in terms of overfeeding time, degree of overfeeding and macronutrient composition. A 3-day overfeeding study in which 21 subjects (15 males, 6 females) consumed 50% more calories than baseline requirements (energy breakdown: 20% protein, 30% fat, 50% carbohydrate) showed GLP-1 was unchanged over the duration of the study [35]. Similarly, Brons *et al*. overfed 26 healthy Danish young men 50% more calories than required (60% fat, 32.5% carbohydrates and 7.5% protein) utilizing a 5-day high fat diet and found no significant change in fasting circulating GLP-1 [33]. A small study on nine lean Caucasian males also found no significant difference in GLP-1 concentration after an overfeeding period which ceased when 5% of body weight was gained (average of 35 days, range of 28–43; composition: ~ 50% carbohydrates, 35% fat, 15% protein) [34]. The excess calories were provided by a liquid drink which was used to bring total caloric intake to a value of 1.4 times the eucaloric diet. Evidently these studies differ in regards to subject homogeneity, length and amount of energy surplus. The negative results from all three studies could be due to the small sample size and/or shorter period of overfeeding (first two studies). The present investigation, utilizing a fairly homogenous sample population of young healthy men from the Newfoundland population, observed a significant increase in serum GLP-1 after a 7-day overfeeding challenge consistent with a typical North American diet (50% carbohydrates, 35% fat, and 15% protein). We suggest that the increase in GLP-1 was a homeostatic protective mechanism to offset the metabolic disturbance caused by the energy surplus.

Previous studies have suggested that obese individuals have a lower GLP-1 secretion as compared to lean individuals [20,24,25,41]. In our cohort, we found no significant difference in fasting GLP-1 concentration between overweight/obese and normal weight subjects. In the entire cohort we found no significant relationship between baseline GLP-1 concentration and markers of adiposity including BMI and percent body fat. However, in the overweight/obese group we found baseline GLP-1 correlated with percent gynoid fat. In general, women are more likely to have greater gynoid fat distribution, and having this distribution is thought to oppose cardiovascular diseases through more efficient fat storage/lipoprotein lipase functionality [42,43]. Additionally in the overweight/obese group baseline GLP-1 concentrations correlated with a negative change in percent gynoid fat. In other words, individuals with higher GLP-1 concentration had a smaller change in percent gynoid fat after the positive energy challenge; therefore the finding suggests higher baseline GLP-1 predicted a reduced gain in percent gynoid fat. Moreover, change in GLP-1 was negatively associated with a change in gynoid fat within the normal weight group, again proposing a protective effect of GLP-1. Nevertheless, with lack of large-scale studies assessing GLP-1 and overfeeding we cannot fully elucidate the predictor ability of baseline/change in GLP-1 on change in metabolic variables.

In this study we also observed the relationships between GLP-1 and markers of lipid metabolism and insulin resistance. At baseline when controlling for percent gynoid fat, GLP-1 was positively correlated with triacylglycerols and markers of insulin resistance and negatively correlated with HDL cholesterol, in the overweight/obese group. Thus taken together, overweight/obese subjects with higher circulating baseline GLP-1 have a less favourable lipid profile (higher triacylglycerols, lower HDL cholesterol) and higher insulin resistance (increased HOMA-IR, HOMA-β, and insulin). This being said, studies have shown administration of GLP-1 receptor agonists to be associated with a beneficial change in lipid profile and insulin resistance [44-47]. Still however, a study by de Luis *et al*. [48] found that after biliopancreatic diversion surgery in morbidly obese patients, basal GLP-1 was negatively associated with HDL-c, consistent with our findings. Additionally it has been found that higher circulating GLP-1 in subjects with metabolic syndrome, are at greater risk for cardiovascular disease [49]. When we observed the relationship between percent change in GLP-1 and percent change in triacylglycerols, a positive association was found in both the normal weight and overweight/obese cohorts (also change in cholesterol was positively correlated with change in GLP-1 in the overweight/obese cohort). We theorize that the increased GLP-1 is trying to compensate for the increase in both triacylglycerols and total cholesterol. However, because our study is a forced overfeeding intervention (subjects ate 70% more calories than required a day) the metabolic disturbance caused by overfeeding potentially overwhelmed GLP-1’s potential effect. Nevertheless more studies are necessary to elucidate the role of GLP-1 in lipid/glucose metabolism.

A limitation of this investigation is that only young men (age 19–29) of Newfoundland descent were studied for one week; therefore similar studies are needed in females, and subjects of different age ranges and ethnic groups. Further large-scale overfeeding studies assessing such cohorts are warranted to fully elucidate the role of GLP-1 during a positive energy surplus. Additionally, only total human GLP-1 was measured rather than the suggested active form, GLP-1 (7–36 amide). Active GLP-1 has a very short half-life and is found in low concentrations, before it is degraded by DPP-IV, while total GLP-1 gives an indication of the secretion from intestinal L-cells. However, total GLP-1 has been shown to positively correlate with active GLP-1 (7-36amide) concentration [50]. Furthermore, although macronutrient composition was relatively constant, we did not account for the composition of fat (polyunsaturated, saturated, etc.) or carbohydrate (complex vs. simple sugars).

## Conclusion

Overall, our study investigated the response of fasting GLP-1 concentration to a 7-day overfeeding protocol in a total of 72 young men from the Canadian province of Newfoundland. In response to the short term energy surplus, circulating GLP-1 significantly increased in the entire cohort, regardless of adiposity. We suggest that the increased GLP-1 may act as a protective mechanism to counteract the positive energy challenge. Additionally at baseline, there was no significant difference in fasting GLP-1 concentration between the lean and overweight/obese groups. However at baseline, GLP-1 was positively correlated with triacylglycerols and markers of insulin resistance, and negatively associated with HDL-c in overweight/obese individuals. Also in this group, baseline GLP-1 was negatively associated with percent change in percent gynoid fat. Percent change in GLP-1 was associated with percent change in specific variables of lipid metabolism (triacylglycerols, total cholesterol) in the overweight/obese group. Although the positive relationship between percent change in triacylglycerols and percent change in GLP-1 was present in the normal weight group, a negative relationship existed between percent change in gynoid fat and percent change in GLP-1. Our results suggest that GLP-1 can potentially serve as a protective factor in obesity and it is involved in lipid/glucose metabolism and fat distribution.

## Competing interests

None of the authors had a personal or financial conflict of interest.

## Authors’ contributions

The author’ responsibilities were as follows: DW: wrote the paper. DW, FC and YY: analyzed the data and performed statistical analysis. DW, FC and PA: assisted with data collection. ER: assisted with GLP-1 measurements. SV assisted with insulin measurements. GS was responsible for the study design, final content, and takes responsibility for the integrity of the data and the accuracy of the data analysis. GS, JC, ER, SV, and YY: assisted with the revisions of the manuscript. All authors read and approved the final manuscript.
